# Research hotspots and trends in robot-assisted surgery in pediatric urology: a bibliometric review based on CiteSpace visual analysis

**DOI:** 10.3389/fped.2026.1652856

**Published:** 2026-01-16

**Authors:** Jingyi He, Diyi Luo

**Affiliations:** 1 Department of Pediatric Urinary Disease Center Nursing, West China Second University Hospital, Sichuan University, Chengdu, Sichuan, China; 2 Key Laboratory of Birth Defects and Related Diseases of Women and Children (Sichuan University), Ministry of Education, Chengdu, Sichuan, China

**Keywords:** pediatric urology, robot-assisted surgery, bibliometrics, CiteSpace, visualization, review

## Abstract

**Objective:**

This study aims to analyze the global research landscape of robot-assisted surgery in pediatric urology, identify developmental trends through visualization methods, and provide references and recommendations for future research directions.

**Methods:**

A retrospective bibliometric analysis was conducted using literature retrieved from PubMed and Web of Science Core Collection, covering the period from January 2005 to March 2025. CiteSpace 6.4.R1 software was used for data processing and visualization. Analyses included publication trends, keyword co-occurrence, keyword clustering, and burst detection to construct knowledge maps and explore research dynamics.

**Results:**

A total of 498 publications were included in the analysis. The number of publications showed a clear upward trend. The author collaboration network was relatively dense, with an identifiable core research team. The University of Chicago emerged as the leading institution in terms of publication volume in the field of robot-assisted surgery in pediatric urology. Gundeti MS was the most productive author, contributing 51 published papers to the field. Keyword clustering analysis revealed 10 clusters, and burst detection identified 20 keywords with significant citation bursts.

**Conclusions:**

Current international research primarily focuses on disease classification and surgical techniques in robot-assisted surgery in pediatric urology. Future studies should continue to explore the clinical potential of robot-assisted surgery, broaden its application scope, and promote the diversified development of pediatric urology.

## 1 Introduction

Robot-assisted surgery (RAS) represents a significant advancement in the evolution of minimally invasive surgery techniques, enhancing surgical precision and elevating operative procedures to a new technological standard. Initially introduced in clinical practice in the United States (U.S.) (1), surgical robots were adopted in China in 2007 and have since been widely and effectively applied in fields such as adult urology (2). RAS is characterized by a high-definition optical imaging system and a highly flexible operating mechanism (3), offering several advantages including shortened hospital stays, reduced pain, improved cosmetic outcomes of the incision, and a lower incidence of postoperative complications (4). However, due to challenges such as limited instrument size, the wide age range of pediatric patients, and physiological and anatomical differences from adults, the application of RAS in the pediatric field has developed relatively slowly (5). In recent years, research interest in pediatric urologic RAS has been steadily increasing (6–8). Nevertheless, no comprehensive bibliometric analysis has yet been conducted to systematically summarize the current research landscape, key focus areas, and emerging trends in this field from a macro-level perspective.

Knowledge graphs (KGs) typically uses entities and relationships to model real-world concepts and their interconnections. Through visual graph representations, KGs can effectively illustrate the core structure, historical development, emerging frontiers, and overall knowledge architecture of a discipline, thereby promoting interdisciplinary integration. As a new analytical approach, KGs provide practical and valuable insights for academic research (9, 10). CiteSpace is a software based on co-citation analysis theory and pathfinding network algorithms, used for bibliometric and visual analysis of scientific literature in specific research domains (11). This study uses the CiteSpace version 6.4.R1 software to conduct a visual bibliometric analysis of global literature on RAS in pediatric urology over the past 20 years, with the aim of providing valuable references and strategic guidance for future research directions and evidence-based decision-making.

## 2. Materials and methods

### 2.1 Study design and ethics

This retrospective bibliometric analysis focused exclusively on published articles and did not involve any human participants or clinical trials. Therefore, ethical approval from an institutional review board was not required.

### 2.2 Data collection

Literature searches were conducted in PubMed database and Web of Science Core Collection database using advanced search strategies. The search scope was limited to publications classified as “Article” and “Review”, and restricted to the English language. The following subject terms were used: “robotic-assisted surgery” OR “robot-assisted laparoscopic” OR “robot-assisted laparoscopic ureterocalicostomy” OR “robot-assisted laparoscopic pyeloplasty (RALP)” OR “robot-assisted laparoscopic ureteral reimplantation” OR “robotic-assisted laparoscopic partial nephrectomy” OR “robotic ipsilateral UU” OR “robotic partial nephrectomy” AND “Children” OR “Toddlers” OR “Pediatrics” OR “Infants” OR “Adolescents.” The publication date range was set from January 1, 2005 to March 10, 2025.

### 2.3 Study inclusion and exclusion criteria

Inclusion criteria: (1) Publications focusing on RAS in pediatric urology that are publicly available. Exclusion criteria: (1) Studies not clearly involving pediatric and urologic RAS; (2) Conference abstracts, announcements, news reports, and other non-research content; (3) Incomplete announcements; (4) Duplicate publications; (5) Publications not in English. The literature screening process was independently conducted by two researchers. In cases of disagreement, a third researcher was consulted to reach a consensus.

### 2.4 Analytical methods

Literature from Web of Science Core Collection was exported in plain text format, while the literature from PubMed database was exported in NBIB format. Both files were saved as download_.txt and subsequently imported into CiteSpace 6.4.R1 for format conversion and deduplication before analysis. The Time Slicing was set from January 2005 to March 2025, with Years Per Slice set to 1. For Pruning, the options Pathfinder, Pruning sliced networks, and Pruning the merged networks were selected. The selected Node Types included Author, Institution, Country, and Keyword, while all other threshold parameters were kept at their default settings. In the resulting visual maps, the node size represents the co-occurrence frequency, node color indicates the corresponding year, and the lines between nodes represent collaborative relationships.

## 3 Results

### 3.1 Bibliometric analysis of publication years

**Figure 1:**
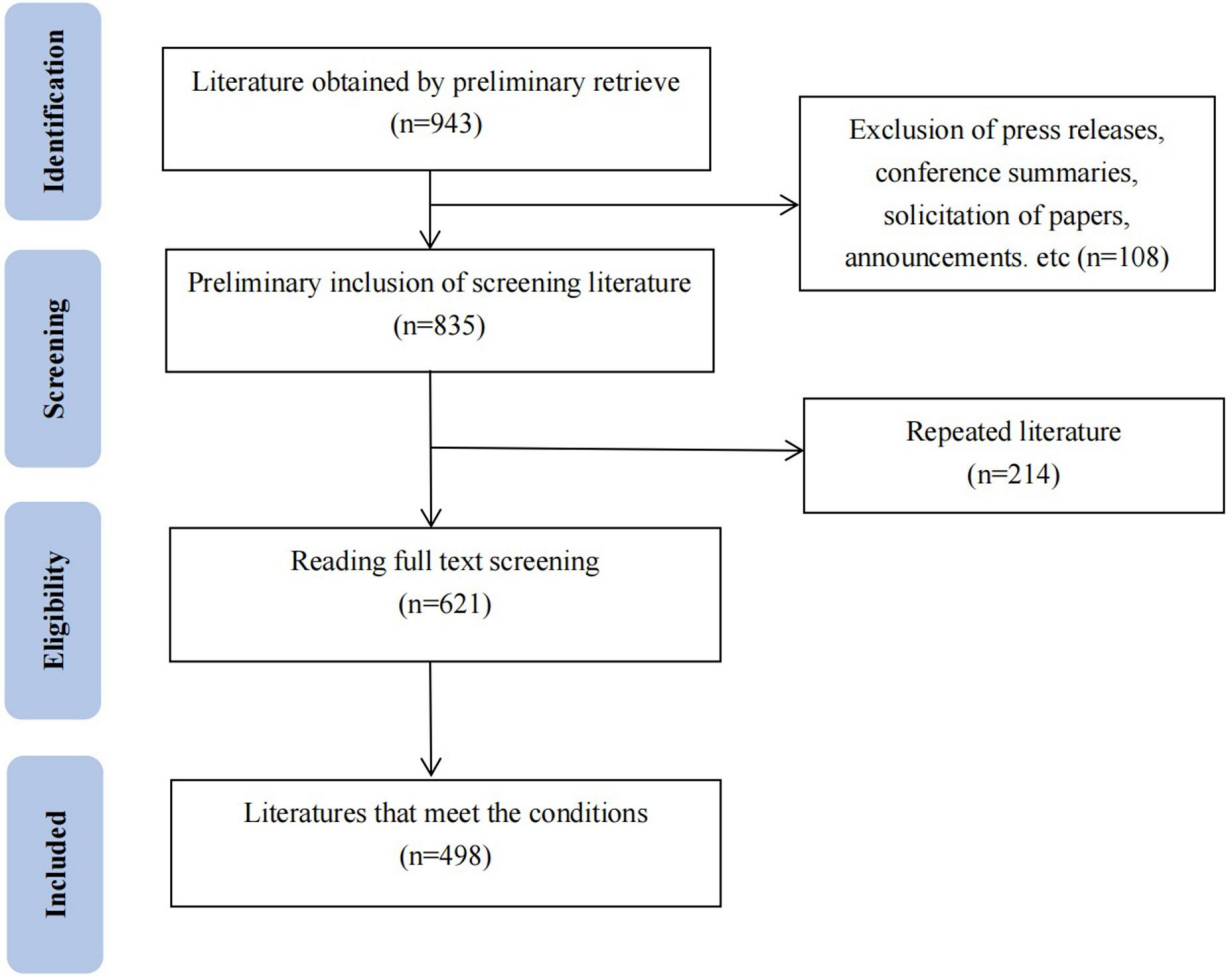
PRISMA flow diagram of the literature selection process. The flowchart illustrates the identification, screening, eligibility assessment, and final inclusion of publications related to robot-assisted surgery in pediatric urology, detailing the number of records retrieved, excluded, and included at each stage.

**Figure 2:**
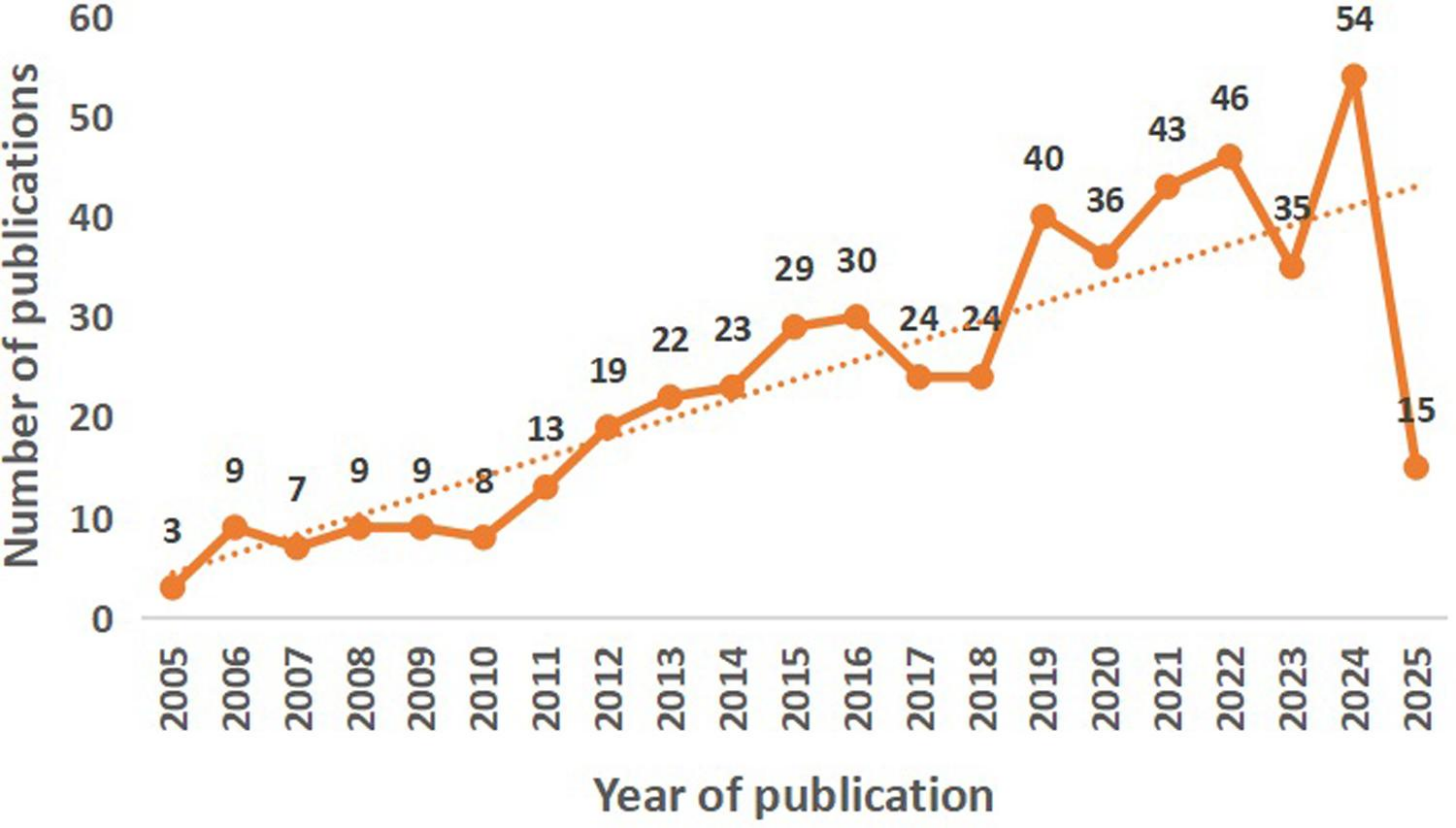
Annual publication trends in robot-assisted surgery in pediatric urology (2005–2025). The line chart shows the yearly number of publications over time, with a fitted trend line indicating the overall growth trajectory of research output in this field.

A total of 943 articles were initially retrieved, and after applying the inclusion and exclusion criteria, 498 articles were included in the final analysis. The literature selection process is shown in Figure 1. Before 2010, the annual publication volume was relatively low, with fewer than 10 articles per year. From 2011 to 2016, a gradual increase was observed. Notably, there was a significant surge in publication volume during the periods 2018–2019 and 2023–2024. Overall, the number of publications has shown a steadily increasing trend, indicating growing academic interest in this field. The publication trend over the past 20 years in RAS in pediatric urology is illustrated in Figure 2.

### 3.2 Bibliometric analysis of institutions

Author affiliation information was recorded according to the institutional affiliation listed in each publication at the time of article submission/publication. As shown in Table 1, the institutions that have published ten or more articles are comprised mainly of academic and medical centers in the United States, Italy, and France. A node centrality greater than 0.1 indicates that the institution occupies a key position in the collaboration network. Higher centrality value suggest closer and more extensive collaborative relationships with other institutions.

**Table 1:** Research institutions with a publication record of at least 10 papers.

Rank	Institutional affiliations of the published articles	Number of publications	Centrality
1	University of Chicago	47	0.11
2	Harvard University	41	0.06
3	University of Pennsylvania	34	0.11
4	Baylor College of Medicine	21	0.21
5	University of Naples Federico II	17	0.01
	Azienda Ospedaliera Universitaria Meyer	17	0.03
6	University of Florence	15	0.04
	Cincinnati Children's Hospital Medical Center	15	0.10
7	Hospital Necker-Enfants Malades	12	0.11
	Seattle Children's Hospital	12	0.19
8	University of Virginia	11	0.09
9	Children's Healthcare of Atlanta	10	0.05

### 3.3 Bibliometric analysis of authors

**Figure 3:**
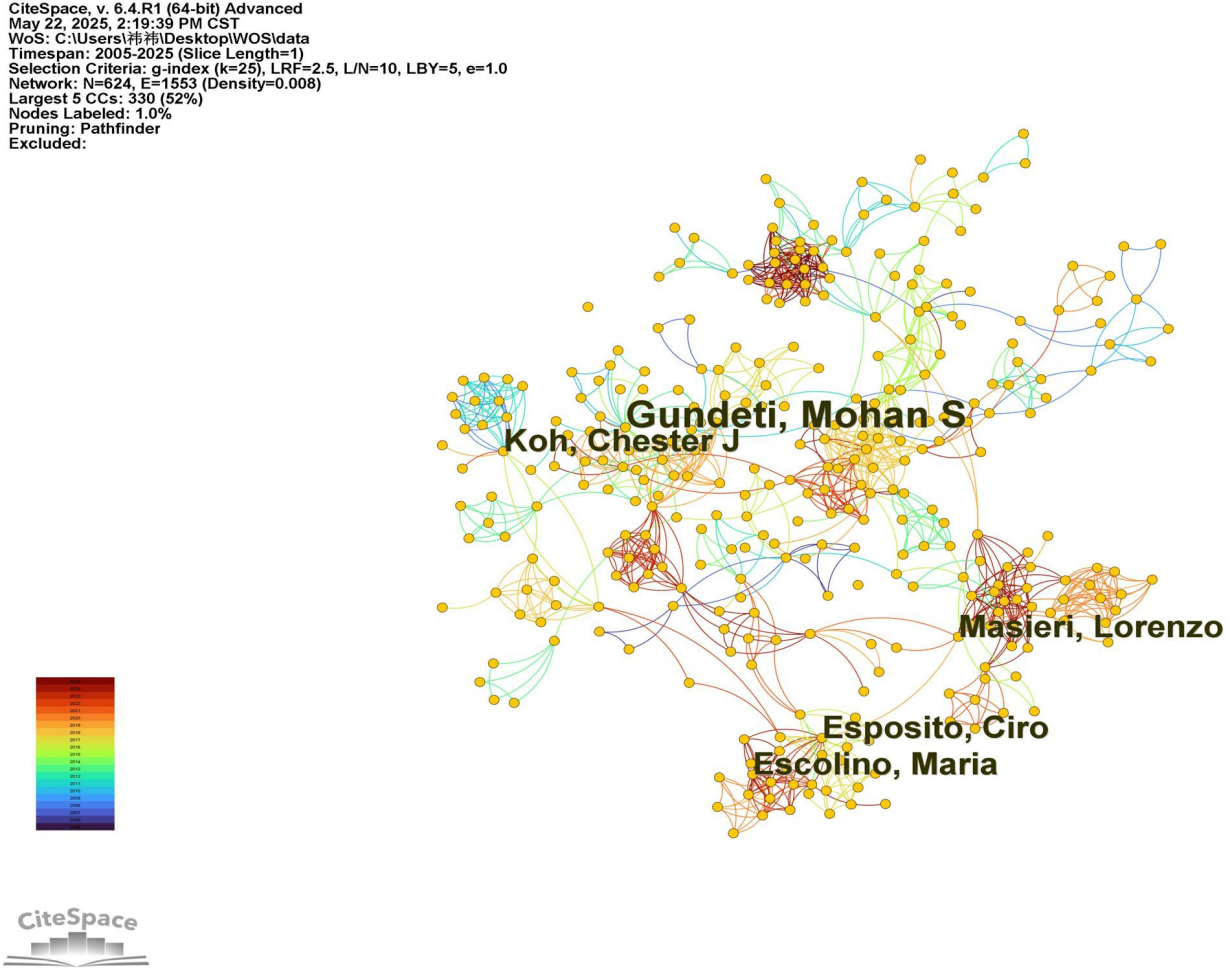
Co-authorship network of authors in robot-assisted surgery in pediatric urology. The network visualization generated by CiteSpace displays collaborations among authors. Node size represents publication frequency, while links indicate co-authorship relationships. Node colors correspond to the time of publication.

The top 10 most productive authors are listed in Table 2. The number of core authors was calculated using Price's Law (12) based on the formula: *N* = 0.749 × √Npmax, where Npmax represents the number of publications by the most prolific author. Authors with more than N publications are defined as core authors. Among the 498 included articles, there were a total of 624 authors. The author with the highest number of publications was Mohan S. Gundeti from the University of Chicago, with 51 publications. Therefore, *N* = 0.749 × √51 ≈ 6. Consequently, 53 authors with more than 6 publications were identified as core contributors. The author collaboration network is shown in Figure 3. Several core research teams have formed in this field, with key representatives including Mohan S. Gundeti, Ciro Esposito, Chester J. Koh, Maria Escolino, and Lorenzo Masieri. These teams have established strong collaborative networks both within and across groups, indicating a robust academic cooperation in this area of study.

**Table 2:** Top 10 authors in terms of number of publications.

Rank	Author	Institutional affiliations of the published articles	Number of publications
1	Gundeti, Mohan S	University of Chicago	51
2	Esposito, Ciro	Federico II University Hospital	25
3	Koh, Chester J	Texas Children's Hospital	24
4	Escolino, Maria	University of Naples School of Medicine	24
5	Masieri, Lorenzo	Meyer Children Hospital	23
6	Peters, Craig A	Texas Children's Hospital	18
7	Blanc, Thomas	Necker Children Hospital	18
8	Nguyen, Hiep T	Banner Desert Children's Hospital	18
9	Casale, Pasquale	Children's Hospital of Philadelphia	16
10	Passerotti, Carlo C	University of São Paulo School of Medicine	15

### 3.4 Bibliometric analysis of keyword co-occurrence

**Figure 4:**
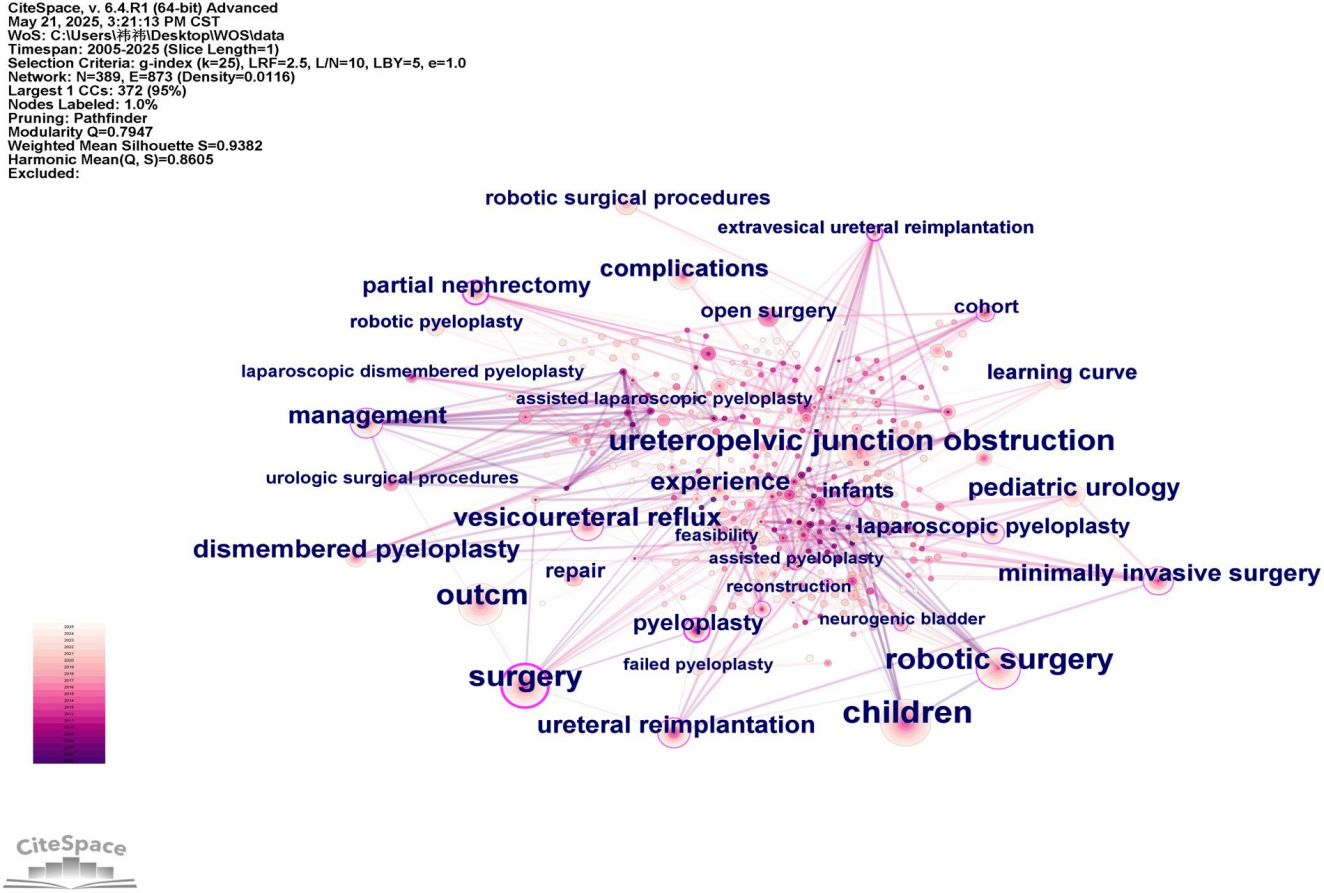
Keyword co-occurrence network in robot-assisted surgery in pediatric urology. This network map illustrates the relationships among high-frequency keywords. Larger nodes indicate higher occurrence frequency, and links represent co-occurrence strength. Colors reflect the temporal evolution of research topics.

Keyword co-occurrence analysis is a statistical method used to analyze the frequency with which keywords appear together to reveal the intensity of their associations and identify research hotspots (13). The frequency and distribution of keywords reflect the current state of the research field and emerging trends. In the co-occurrence network, larger nodes represent keywords with higher frequencies, indicating greater academic attention (14). These keywords indicate that the current research in RAS in pediatric urology mainly focuses on diseases types and postoperative outcomes. The keyword co-occurrence network consisted of 389 nodes and 873 links, with a network density of 0.0116, as shown in Figure 4.

### 3.5 Bibliometric analysis of keyword clustering

**Figure 5:**
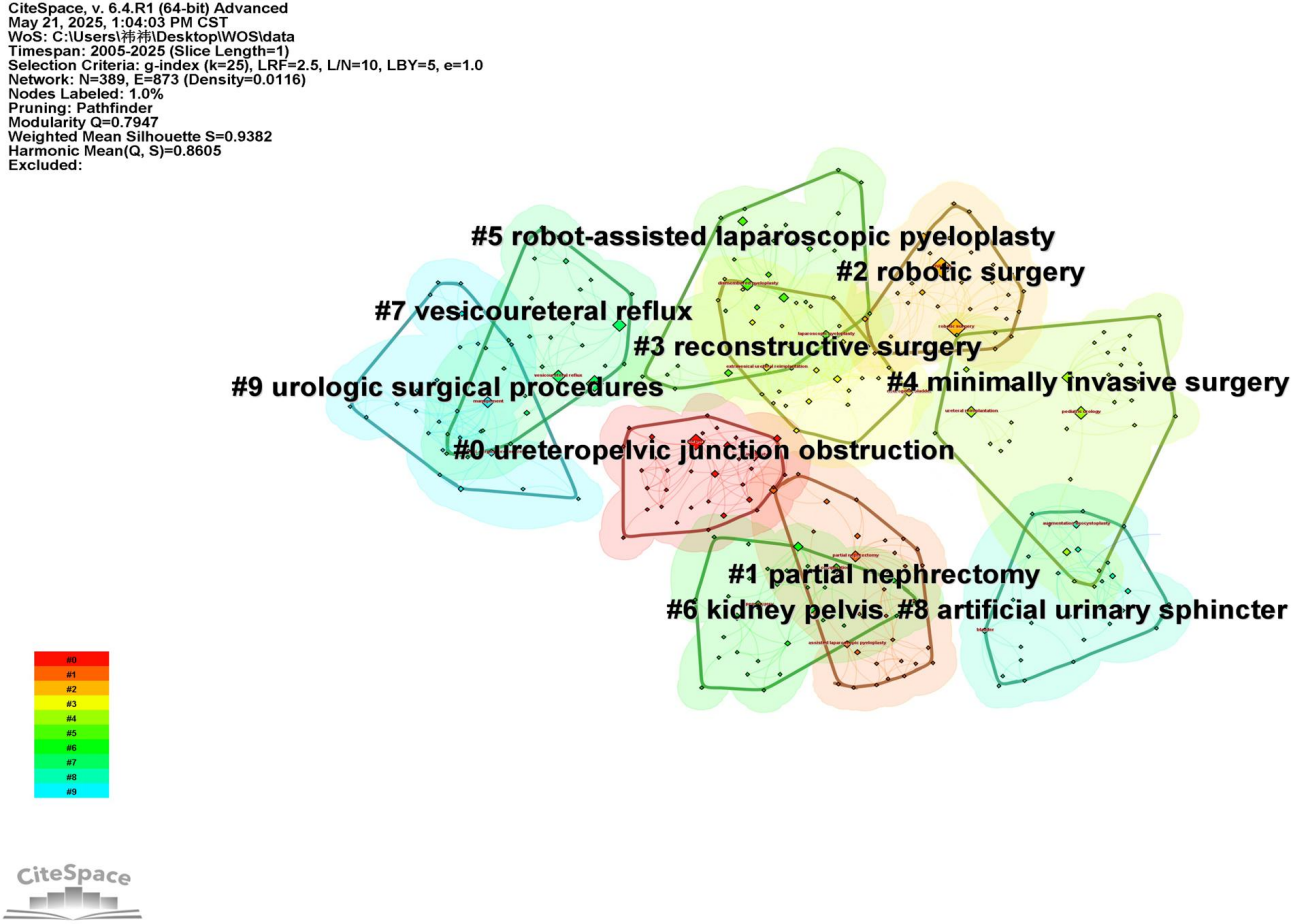
Keyword clustering analysis based on CiteSpace. The clustered network identifies major research themes in robot-assisted pediatric urology. Each cluster is labeled with representative keywords, and the colors distinguish different thematic groups, reflecting the knowledge structure of the field.

To further explore the thematic structure of the research field, a clustering analysis was conducted on the keyword co-occurrence network, resulting in ten major clusters, as shown in Figure 5 and Table 3. The modularity of the network is quantified by the Modularity Q index, which ranges from 0 to 1. A Q value of greater than 0.3 indicates a significant clustering structure within the network, while values closer to 1 suggest more distinct and well-separated clusters, reflecting better clustering performance. Silhouette S is a contour coefficient used to measure network homogeneity, with values ranging from −1 to 1. A silhouette value greater than 0.7 indicates strong clustering with high homogeneity. As shown in Figure 5, the keyword clustering analysis yielded 10 keyword clusters with a Modularity Q value of 0.7947 and a Silhouette S of 0.9382, indicating a well-defined modular structure with excellent homogeneity and clustering validity. As shown in Figure 5, these clusters can be broadly grouped into the following thematic categories: ① Disease types: Clusters #0 and #7 ② Surgical sites: Clusters #1, #5, #6, and #8 ③ Surgical approaches: Clusters #2, #3, #4, and #9. There is a certain degree of overlap between the clusters, indicating close and relevant connections among different research areas. The Size value listed in Table 3 refers to the number of publications within each cluster, while the Silhouette value indicates the degree of internal cohesion—the higher the value, the greater the homogeneity within that cluster.

**Table 3:** Keyword clustering table.

Cluster ID	Label (LLR)	Size	Silhouette	Mean (year)	Included keywords
#0	Ureteropelvic junction obstruction	33	0.904	2015	Augmentation; retrocaval ureter; urachal cyst; anomaly
#1	Partial nephrectomy	32	0.922	2017	Nephron-sparing surgery; minimally invasive surgeries (mis); renal duplication; surgical outcomes
#2	Robotic surgery	31	0.910	2015	Learning curve; pediatric surgery; da vinci; pediatric kidney transplantation
#3	Reconstructive surgery	31	0.915	2012	Transperitoneal; heminephrectomy; duplex renal anomaly; ureteral reimplantation
#4	Minimally invasive surgery	31	0.936	2016	Systematic review; ectopic ureters; duplex system; open ureteral reimplantation
#5	Robot-assisted laparoscopic pyeloplasty	31	0.887	2016	Pediatric patients; laparoscopic pyeloplasty; flexible endoscopy; robot-assisted laparoscopic ureteral reimplantation
#6	Kidney pelvis	31	0.924	2014	Ureteral obstruction; robotics; toddler; antireflux plasty
#7	Vesicoureteral reflux	29	0.951	2017	Primary obstructive megaureter; ureteral reimplantation; outpatient; day surgery
#8	Artificial urinary sphincter	28	1.000	2016	Robotic-assisted surgery; neurogenic bladder; bladder neck reconstruction; bladder neck sling
#9	Urologic surgical procedures	28	0.981	2011	Diagnosis; vesico-ureteral reflux; urinary bladder; robotic paediatric pyeloplasty

### 3.6 Bibliometric analysis of keywords with citation bursts

**Figure 6:**
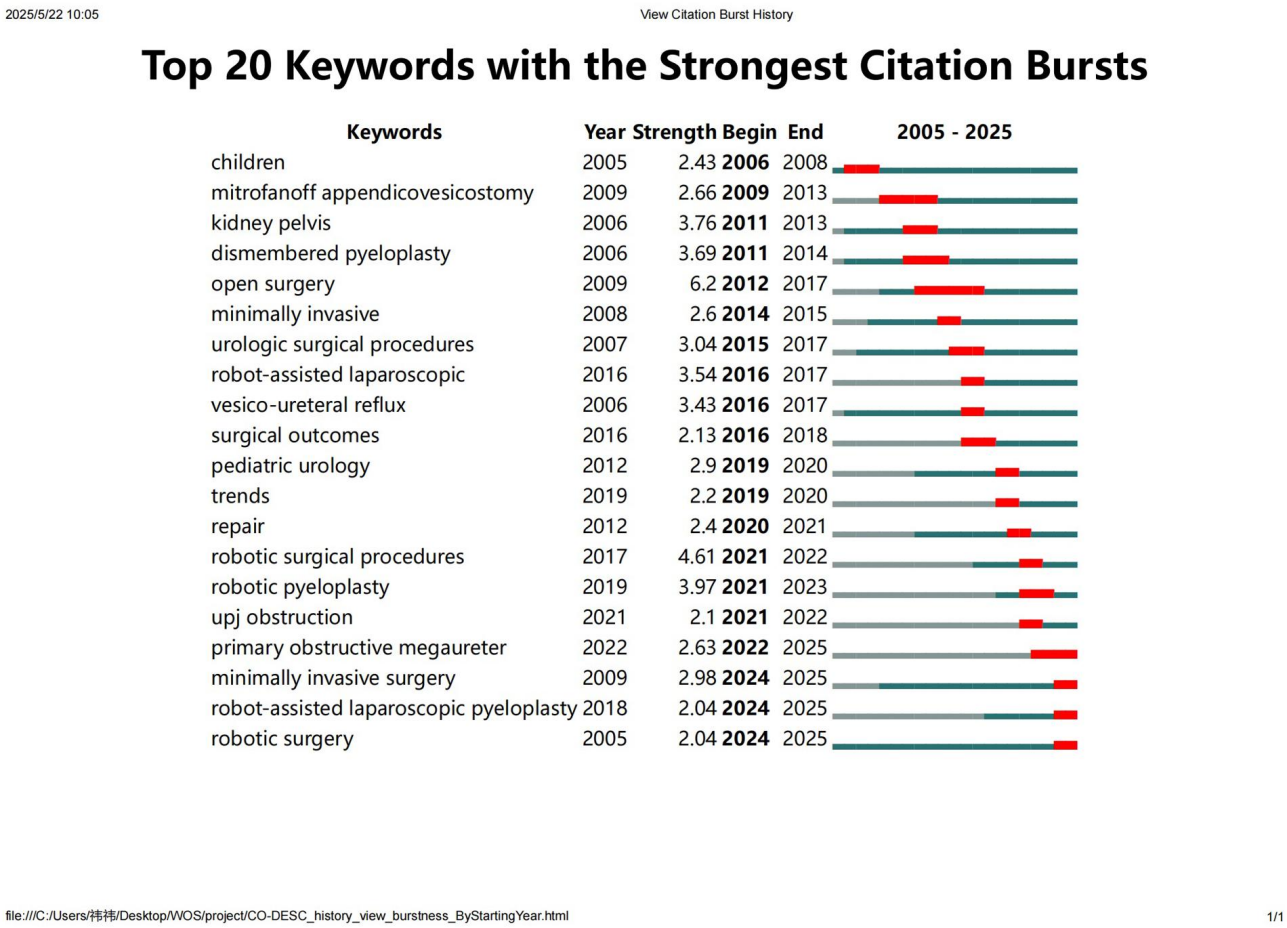
Top 20 keywords with the strongest citation bursts from 2005 to 2025. The burst detection analysis highlights keywords that experienced significant increases in citation frequency over specific periods, indicating emerging trends and research frontiers in robot-assisted surgery in pediatric urology.

Citation burst detection identifies keywords that exhibit a sharp increase in usage frequency over a short time, signaling emerging trends or research frontiers. The year indicates the initial appearance of the corresponding keyword in the literature. The start and end years denote the time span of the burst, and the length of the red line in the visualization corresponds to the duration of the burst period. As shown in Figure 6, research before 2010 mainly focused on the comparison between open and minimally invasive surgery. After 2010, the emphasis shifted toward evaluating the outcomes of RAS. In recent years, increasing attention has been given to the application of robotic techniques in treating conditions such as UPJO and primary obstructive megaureter (POM).

## 4 Discussion

### 4.1 General characteristics

This study is the first to apply CiteSpace to bibliometrically analyze research related to robotic-assisted surgery in pediatric urology. A total of 498 publications were retrieved from PubMed and the Web of Science Core Collection between January 1, 2005, and March 10, 2025. The annual number of publications has shown a steady upward trajectory, indicating that this field is rapidly expanding and continuously gaining academic attention. The distribution of the most prolific institutions (≥10 publications) is detailed in Table 1. Of the twelve centers that meet this criterion, eight are located in the United States, while three and one are from Italy and France, respectively. The University of Chicago leads with 47 publications (9.4%), highlighting its dominant contribution. As the birthplace and primary application market of surgical robotic systems, the United States benefits from first-mover advantage, strong industry–academia integration, and high-volume pediatric centers supported by stable funding and specialized research infrastructure (15). These structural advantages have facilitated early adoption, rapid technical maturation, and sustained research productivity. Similarly, several European countries demonstrate strong publication output, which may reflect differences in resource allocation, investment capacity, regulatory environments, and timing of technological diffusion across regions. Strengthening global academic collaboration and knowledge exchange may therefore support more equitable dissemination and effective implementation of pediatric robotic surgery internationally.

Despite these advances, evidence gaps persist. Most current clinical studies remain single-center, observational, and short-term in nature, and there continues to be a lack of large-scale comparative outcome studies, rigorous cost-effectiveness analyses, and long-term functional outcome evaluation. Future studies should emphasize multicenter collaboration, standardized outcome frameworks, and prospective long-term follow-up to generate higher-level clinical evidence that can better inform value-based decision-making.

Beyond mapping research structure, these bibliometric findings also provide important clinical and translational insight. High-output countries and institutions may serve as reference models to support global capacity-building, competency-based training, and standardized robotic curriculum development in pediatric urology. Moreover, observed publication patterns may assist policymakers in rational resource allocation and in promoting more equitable access to robotic platforms worldwide. Advancing research beyond technical feasibility toward comparative effectiveness trials, economic evaluation, and long-term functional outcome assessment will be essential to accelerate evidence-based and sustainable implementation of robotic surgery in pediatric urology.

### 4.2 Research topics and emerging trends

Keywords reflect the core thematic structure of published studies and serve as an effective indicator of evolving research priorities. The keyword co-occurrence and clustering analyses in this study demonstrate that research on pediatric robotic-assisted surgery over the past two decades has been mainly concentrated in hydronephrosis-related conditions (UPJO and VUR), renal duplication anomalies, urachal cysts, and neurogenic bladder (NB). Correspondingly, procedure-related hotspots have been centered on RALP, robotic partial nephrectomy, nephron-sparing surgery, pediatric kidney transplantation, ureteral reimplantation, and artificial urinary sphincter–related interventions. These patterns reveal that the research focus has gradually shifted from early adoption of robotic platforms in relatively standardized reconstructive procedures toward broader expansion into more complex anatomical corrections and functional reconstructive domains.

Neurogenic bladder (NB) also represents a growing niche within this research landscape. In addition to procedures such as artificial urinary sphincter implantation, bladder augmentation, and ureteral reimplantation, two studies have reported rare yet innovative robotic bladder neck reconstruction techniques in pediatric patients (robotic bladder neck plication and a robotic-assisted “keel” reconstruction approach), suggesting that novel reconstructive innovations may continue to emerge in this space (16, 17). The accumulation of such specialized indications indicates a potential future trajectory toward increased technical diversification and refinement within pediatric robotic urology.

Overall, these keyword-based patterns reflect an ongoing evolution from feasibility-focused adoption toward procedure expansion, surgical complexity escalation, and innovation-driven refinement. As robotic capabilities continue to improve, future research trends are likely to further integrate advanced reconstructive techniques, broaden indication portfolios, and develop standardized frameworks that support the safe and evidence-based implementation of robotic platforms in pediatric urology worldwide.

## 5 Limitations

Our study has several limitations that should be taken into account when interpreting the findings. First, the data sources were restricted to PubMed and Web of Science. Although these are widely recognized and authoritative databases, the exclusion of other major sources such as Embase, Scopus, and regional or non-English journals may introduce database coverage bias and lead to incomplete capture of the global research output. Second, the bibliometric analysis relied on keyword-based retrieval, which is inherently sensitive to the selection of search terms and may be affected by language bias and terminology evolution over time. This may result in the omission of relevant studies that apply alternative expressions. Third, the interpretation of network visualization and cluster relationships may still involve subjective judgment. Although CiteSpace includes multiple algorithms to enhance structural robustness, discrepancies among them may affect the stability and consistency of the identified knowledge structures. Collectively, these factors may influence the comprehensiveness and interpretability of our findings.

## 6 Conclusion

These findings also carry important clinical implications, underscoring the need to strengthen global collaboration, improve equity in resource allocation, and accelerate capacity-building in regions with emerging robotic adoption. Prioritizing multicenter comparative outcome research, cost-effectiveness evaluation, and long-term functional follow-up should be considered essential future directions to support evidence-based and sustainable implementation of pediatric robotic surgery worldwide.
